# Assessing physical-exercise suitability in an urban canal corridor: evidence from the Cangzhou reach of China’s Grand Canal

**DOI:** 10.3389/fpubh.2026.1885101

**Published:** 2026-08-12

**Authors:** Xin Zhang, Wenjun Zhong, Yi Yu, Lei Cao

**Affiliations:** 1 School of Architecture & Art Design, Hebei University of Technology, Tianjin, China; 2 Anhui Economic Press and Media Co., Ltd., Hefei, China; 3 School of Architecture and Urban Planning, Chongqing University, Chongqing, China; 4 School of Architecture, Tianjin University, Tianjin, China

**Keywords:** Cangzhou section of the Grand Canal, evaluation study, expert consultation—analytic hierarchy process, physical-exercise suitability, river channel space

## Abstract

**Introduction:**

River corridors along the Grand Canal provide potential settings for outdoor physical exercise; however, existing assessment tools do not sufficiently address the combined requirements of longitudinal continuity, waterside accessibility, safety, comfort, and heritage-sensitive spatial conditions in linear canal corridors.

**Methods:**

This study assessed 13 sample sites within a 24-km urban reach of the Grand Canal in Cangzhou, China. Based on field surveys, questionnaires, expert consultation, and the analytic hierarchy process (AHP), an evaluation system was developed from four criteria: continuity, accessibility, safety, and comfort. Eighteen indicators were established and applied to 13 sample spaces.

**Results:**

The results showed that safety carried the highest weight, followed by accessibility, while continuity and comfort had relatively lower weights. Using a consistent hierarchical AHP procedure and treating the site as the primary unit of aggregation, the mean composite suitability score of the 13 surveyed sites was 3.488, corresponding to Grade III (moderate). The west bank scored slightly higher than the east bank (3.545 vs. 3.422). Among spatial types, park-type spaces performed best (4.441, Grade II), followed by cultural-activity-type spaces (3.723, Grade III) and urban-road-type spaces (3.072, Grade III).

**Discussion:**

The study reveals a mismatch between the relatively strong structural continuity of the canal corridor and the insufficient provision of activity-supportive facilities and environmental conditions. The proposed framework serves as a diagnostic tool for identifying priority deficiencies and informing type-specific renewal of urban canal spaces.

## 1 Introduction

As the concept of “prevention first,” as articulated in policy documents ranging from the 1995 Outline of the National Fitness Programme to the 2012 Strategic Research Report on “Healthy China 2020,” has become increasingly embedded in public consciousness, exercise and fitness have been strongly promoted as a means of “preventing illness before it occurs.” In 2020, the Opinions on Strengthening the Construction of National Fitness Venues and Facilities and Developing Mass Sports further proposed that non-sports land within new spatial carriers, including parks, green spaces, marginal plots, and fitness trails, should be fully utilized to increase sports and fitness spaces and facilities and better meet public demand (1, 2). Through the intensive issuance of policy documents, the state has responded to public concerns regarding people’s livelihoods, and the National Fitness Programme has become one of the key areas in which the state prioritizes public welfare. Alongside national policy guidance, the public has increasingly attached importance to the cultivation of exercise habits. As a fundamental attribute of human health, health can be enhanced through interaction with specific environments; the benefits people derive do not stem from blue space itself, but from engagement with the specific environment during such interaction (3). Owing to their linear spatial characteristics and unique ecological and cultural resources, riverine spaces have, either actively or passively, become the primary choice of exercise venues for local residents (4). Consequently, in response to the call to build a “Healthy China” and promote national fitness initiatives, exploring the development of sports and fitness venues based on riverine spaces holds considerable theoretical and practical value. Research on the suitability of riverine spaces for sports and fitness not only helps unlock their latent potential, but also addresses the structural contradiction between the public’s growing and diversified demand for such spaces and the relative scarcity of land available for this purpose, thereby carrying significant practical significance.

At present, the development of the Grand Canal Cultural Belt is steadily advancing. Existing research has largely focused on the protection of Grand Canal cultural heritage, ecological restoration, and the integration of cultural and tourism industries, with an emphasis on macro-level spatial planning and top-level design (5–7). However, targeted research on riverine spaces as venues for outdoor fitness remains limited. This not only neglects their core function in meeting the fitness needs of urban residents, but also fails to integrate the regional characteristics of the Grand Canal into an evaluation system for physical-exercise suitability (8, 9). In terms of research methodology, existing suitability assessments predominantly rely on single-dimensional indicator systems, focusing mainly on landscape aesthetics, ecological integrity, and safety (10). Such approaches insufficiently account for the physiological load of fitness participants and the suitability of exercise environments, and they have yet to incorporate quantitative evaluation criteria (11). Furthermore, the surveyed urban Cangzhou reach is characterized by the coexistence of linear corridor continuity, two-bank spatial organization, heterogeneous urban interfaces, and heritage-related management requirements. These characteristics provide an appropriate empirical context for adapting and testing a physical-exercise suitability framework for linear heritage canal spaces (12).

In response to these limitations, this study develops and applies a context-adapted framework for assessing the suitability of urban canal spaces for physical exercise. The study focuses on three questions: first, how the health-supportive characteristics of blue space, longitudinal corridor continuity, and heritage-sensitive spatial conditions can be incorporated into a multidimensional assessment framework; second, which spatial dimensions and indicators are considered most important by experts and users; and third, how suitability and priority deficiencies vary among park-type, urban-road-type, and cultural-activity-type canal spaces. The study makes three contributions. First, it reframes the health value of canal blue space as a question of usable spatial conditions rather than the mere presence of water. Second, it adapts generic public-space assessment criteria to the longitudinal continuity, two-bank organization, water-edge risks, and heritage-sensitive interfaces of a linear canal corridor. Third, it provides a type-sensitive diagnostic approach that distinguishes the renewal priorities of park-type, urban-road-type, and cultural-activity-type canal spaces.

## 2 Literature review and research definition

### 2.1 Literature review

#### 2.1.1 The Cangzhou section of the Grand Canal

Existing research on the Grand Canal can be broadly grouped into four main themes: the relationship between the Grand Canal and the development of cities along its route, the conservation of Grand Canal heritage, the transmission of Grand Canal culture, and the optimization of cultural and tourism industries related to the Grand Canal. This body of research includes both theoretical investigations and practical applications. Research on the Cangzhou section has mainly focused on the value, conservation, utilization, and development of cultural heritage (13). Common approaches include tracing the historical evolution of the Grand Canal, summarizing patterns of spatiotemporal change, and examining low-impact cultural and tourism development (7, 14), as well as proposing improvements and upgrades from specific perspectives. For example, Chen Qiujing used the Cangzhou section as a case study and traced the evolutionary process and value characteristics of the Ming- and Qing-dynasty Grand Canal from the perspective of cultural routes (15). Chai defined the concept of “non-preferred zones” for canal resources in the Cangzhou section and explored potential pathways for tourism development (16). Liu conducted design research on the riverside landscape of the Grand Canal’s Cangzhou section from a regional cultural perspective (17), introduced environmental assessment methods in her study of the landscape renovation of the Cangzhou Canal (18).

The Cangzhou reach provides a relevant empirical setting because its urban canal corridor combines several spatial conditions that are not usually present simultaneously in bounded parks. The surveyed reach extends along both banks, is repeatedly segmented by bridges and urban roads, and contains pronounced transitions among park-type, road-adjacent, and cultural-activity spaces. Its meandering watercourse also produces uneven relationships between the canal, adjoining urban land, pedestrian routes, and activity nodes. Meanwhile, the corridor must accommodate multiple functions, including flood-control infrastructure, everyday recreation, physical exercise, landscape use, and the conservation and presentation of canal heritage. These overlapping spatial demands make the area suitable for examining the applicability of a context-adapted exercise-suitability framework.

#### 2.1.2 Riverside spaces

Early research on riverine spaces primarily focused on functional requirements such as enhancing navigation capacity, flood control and disaster mitigation, and bank erosion management (19). From the 1980s to the late 1990s, research gradually shifted to the key factors influencing waterfront development, with emphasis on analyzing existing problems, assessing development trends, and proposing optimization strategies. During this period, river channels were widely recognized as urban ecological corridors and a vital component of the urban ecological foundation (20, 21). Related studies also addressed the planning and design of urban waterfront areas, spatial transformation, the provision of leisure and recreational facilities, and the expression of historical and cultural elements (3, 22, 23). Since the early 21st century, the health-promoting and therapeutic value of riverine spaces has received increasing attention. Foley and Kistemann proposed the concept of “blue space,” defining it as a water-centered health-supportive environment with the potential to promote human well-being (24, 25). The European Union’s “BlueHealth” initiative has further developed the BlueHealth Environmental Assessment Tool (BEAT) for evaluating urban blue spaces, thereby driving research on riverine spaces toward a deeper understanding of therapeutic mechanisms, health pathways, and their effects on public health and residents’ well-being (26). Meanwhile, in the context of the growing popularity of preventive healthcare, the potential of riverine spaces for physical exercise and fitness has attracted increasing scholarly attention. Some researchers, beginning with the needs of national fitness, have demonstrated the feasibility, advantages, and characteristics of the “sports + river” model and conducted related design practices (11, 27). Other studies, guided by jogging activities, have evaluated the spatial quality of lakeside walking trails, and, under the concept of exercise prescription, have explored the feasibility of integrating urban waterfront sports spaces with health promotion (28, 29).

#### 2.1.3 Spatial suitability assessment framework

Internationally, spatial suitability assessment has a long research history and a relatively well-established theoretical framework. Related studies span a wide range of fields, including the planning of public activity spaces, the layout of public service facilities, site design, land-use planning, and specialized spatial environments. Over time, this line of research has further expanded to include coupled assessments of physical activity and spatial suitability. Representative assessment frameworks are presented in Table 1.

**Table 1:** Evaluation system for the suitability of representative sports and fitness activities.

Tool	Application scenarios/scope	Developer (s)/date	Content of first-level elements	Number of second-level elements
Recreation facility evaluation tool	Parks, leisure centers, sports grounds and other fitness facilities	(25)	Facilities and amenities, maintenance condition, safety design	69
Bedimo-Rung assessment tools	Sports facilities in the park	(56)	Facility features, environmental conditions, accessibility, aesthetic appeal, safety	181
Environmental assessment of public recreation spaces tool	Public recreational and sports facilities	(57)	Footpaths, garden paths, general areas, water features, catering facilities, amenities, educational/historical features, recreational facilities. 16 in total	646
The physical activity resource assessment	An Evaluation of the Types, Characteristics, Quality and Negative Factors Affecting Sports Facilities	(58)	Resource characteristics, amenities, adverse environmental conditions / disorderly behavior, facilities for activities	63
Parks, activity and recreation among kids	Outdoor recreational spaces for children and young people	(59)	Key features of the park, recreational facilities, amenities, disorderly or unsightly conditions	44

### 2.2 Research gap

The limitations of existing research can be summarized in three aspects. First, studies of the Grand Canal landscape have primarily focused on cultural heritage conservation, ecological restoration, cultural tourism development, and macro-level spatial planning. Comparatively less attention has been paid to how canal-side public spaces support residents’ routine physical exercise or how their exercise suitability can be systematically diagnosed. Although the integrated development of the Grand Canal Cultural Belt, public health, and national fitness has received increasing policy attention, existing research has not yet provided sufficient evidence or practical assessment tools for identifying the exercise-supportive strengths and deficiencies of different canal-side spatial types.

Second, although previous studies have demonstrated the potential physical and mental health benefits of blue spaces, these general health-supportive principles have not been sufficiently translated into operational indicators for assessing everyday physical exercise in linear canal corridors. Existing studies have emphasized the roles of ecological quality, supporting facilities, accessibility, and environmental experience in encouraging outdoor activity, but many assessments remain focused on general environmental quality or the feasibility of integrating sports with waterfront development. Consequently, limited attention has been paid to whether canal environments provide continuous, accessible, safe, and comfortable conditions for sustained physical exercise.

Third, the existing evaluation system lacks specificity and frontier relevance. Current spatial suitability assessments are increasingly moving toward specialization and precision, with a focus on optimizing indicator systems by integrating regional characteristics and functional requirements. Existing international tools such as RFET and BRAT are primarily designed for bounded parks, recreational facilities, and community spaces. They therefore provide limited guidance for assessing the longitudinal continuity, repeated waterside access, water-edge risks, and heritage-sensitive spatial interfaces of linear canal corridors. The proposed framework operationalizes the health-supportive potential of canal blue space through four interrelated dimensions. Continuity concerns whether water-edge routes and footpaths support sustained linear movement. Accessibility concerns whether users can conveniently reach the water, exercise grounds, and supporting facilities. Safety addresses risks associated with water edges, vegetation, paths, exercise equipment, activity grounds, and ancillary facilities. Comfort concerns whether the physical and perceptual conditions of the canal corridor can support prolonged and repeated use. Together, these dimensions assess not the presence of water alone, but whether the canal environment can be safely and continuously used for everyday physical exercise.

In response to these limitations, this study develops a context-adapted diagnostic framework for evaluating physical-exercise suitability in a linear heritage canal corridor. Field surveys, user questionnaires, expert consultation, and AHP are combined to establish indicator weights and assess the performance of the selected sites. The resulting scores are compared across evaluation dimensions and spatial types to identify priority deficiencies and provide differentiated planning implications.

### 2.3 Research definition

#### 2.3.1 The riverside spaces of the Cangzhou section of the Grand Canal

In contrast to general waterfront spaces, the concept of “riverine space” adopted in this paper is defined according to the characteristics of the actual study object, namely the distinct linear river system. The presence of such linear watercourses is a key feature that cannot be overlooked in this study. Drawing on the conclusions of previous scholars, riverine space is generally understood as comprising three components: the water body, the water’s edge, and the adjoining land area (3). River channel space is therefore closely related to waterfront space, and should include surrounding elements such as landscape structures, buildings, garden paths, and vegetation (30). At the same time, as an important transitional interface connecting the water body and urban land, river channel space possesses both public accessibility and ecological service functions, and plays an important role in preserving urban history and culture, facilitating social activities, and enhancing spatial vitality (31). In the context of this study, the riverine space along the Cangzhou section of the Grand Canal is defined as follows: (1) it mainly includes the water surface, embankments, and the embankment crest road, together with roadside greenery and ancillary areas. It exhibits the linear characteristics of the water body to which it belongs, as well as the spatial integrity of both riverbanks, and is therefore classified as a linear waterfront space; (2) centered on the water system, its spatial location lies at the interface between land and water. It is a spatial setting defined by its relationship with surrounding boundaries and consists of a system of multiple interrelated elements (Table 2).

**Table 2:** Classification of river space components.

Type of space	Components	Functionality	Element components
Water surface area	River water	Determining the fundamental approach to the design of riverine spaces	Natural components
	Aquatic plants	An integral part of the ecosystem	
	Riverbed	Enhancing the diversity of riverine habitats to stabilize ecosystems	
	Bridge	A key structural element linking the two sides	Artificial components
	Sandbank	Land formed by the accumulation of silt and sand in a watercourse (not an artificial beach)	Natural components
	Hydraulic engineering	Facilities	Artificial components
Waterfront space	Waterfront walkway	A footpath connecting the waterside areas	Artificial components
	Embankment	An essential component of the watercourse and a key factor in determining its character	
	Quay	Water transport hub	Artificial components
	Waterfront green space	A key aspect in determining the style of the riverfront landscape; the main venue for sporting activities	Natural/ Artificial components
	Landscape features	The visual quality of the space reflects local characteristics	Artificial components
	Dike top road	A pathway connecting the waterfront areas	Artificial components
	Road ancillary facilities	A key foundation for ensuring the quality of riverine spaces	Artificial components
Waterfront Space (Outside the scope of this study)	Landscape architecture	An integral part of creating a distinctive atmosphere, providing people with a variety of functional spaces	Artificial components

#### 2.3.2 Sports and fitness activities

Physical exercise and fitness activities primarily take place in fitness spaces that are functionally and spatially suited to such activities and are influenced by factors such as the surrounding environment, vegetation, and topography (11, 32). In An Analysis of Pathways and Elements in Healthy City Planning, Wang Lan classified physical activity into transport-related, domestic, occupational, and leisure physical activity. The “sports and fitness activities” examined in this study belong to leisure physical activity, with health as their fundamental objective. Through such activities, the standards for exercise-related health established in public health can be met (33). Based on the list of sports and exercise activities issued by the General Administration of Sport of China, and informed by field investigations that comprehensively considered constraints such as actual site conditions, this study has compiled and organized the sports and fitness activities involved in the research (Table 3). In addition, by classifying the characteristics of sports and fitness activities within the sample spaces, the activities were categorized into three types: linear sports and fitness activities, point-dispersed sports and fitness activities, and point-clustered sports and fitness activities (Table 4). Building on this, the characteristics of public exercise and fitness behavior along the Cangzhou section of the Grand Canal are summarized from the dual perspectives of behavioral manifestations and intrinsic motivation, thereby providing a basis for constructing the evaluation framework (Table 5).

**Table 3:** Statistics table of sports and fitness activity types applicable to Cangzhou section canal and river space.

Activity type	Common activity areas	Characteristics of the space
Cruise ship	On the water	Open waters with a gentle current
Skipping stones		
Fishing	By the water	
Bicycle	By the water	Path-based space
Jogging		
Roller skating		
Square dancing	By the water	Flat, open space
Skateboard		
Skipping		
Bounce a ball		
Fitness equipment		
Amusement rides		
Martial arts		
Conversation		
Walking		
Tennis	By the water	Dedicated sports area
Volleyball		
Badminton		
Table tennis		

**Table 4:** Table of classification of sports and fitness activity types.

Classification dimension	Linear fitness activities	Sporadic fitness activities	Localized fitness activities
Features	Activities carried out in path-based spaces	Standalone static activities	Team sports that foster social interaction
Activity type	Jogging, boat trips, brisk walking, strolling, cycling, rollerblading, skateboarding, chatting	Stretching, fishing, skipping stones, skateboarding, skipping, bouncing a ball, chatting, martial arts, shuttlecock,	Square dancing, tai chi, fitness equipment, playground facilities, socializing, tennis, volleyball, badminton, table tennis

**Table 5:** Table of principal behavior characteristics.

Features		Explanation
Sustainability	Common characteristics	The continuity of participants’ exercise habits varies, with each having their own duration of activity.
Suitability		The individual exercises rational control over their level of activity in accordance with different objectives, in order to achieve a state of physical and mental well-being.
Initiative		The individual carries this out of their own accord, driven by their inner needs; it is a spontaneous rather than a passive act.
Safety	Distinctive features	The subject’s avoidance of damaged equipment and unsafe areas.
Normative		All forms of exercise and physical activity have specific movement guidelines, and one must adhere to these guidelines in order to reap the health benefits.
Phased		Exercise and fitness are gradual processes; whether you are aiming to vary your heart rate or increase your intensity, you should always start gently and build up gradually.
Regularity		Regular exercise requires a long-term, sustained commitment before noticeable physical changes occur and health benefits are realized.

#### 2.3.3 Suitability assessment methods

Suitability assessment is conducted based on landscape resources within a specific area and the demands of development and utilization. It examines the suitability and constraints of a given environment from both ecological and social perspectives, thereby classifying levels and grades of suitability (34). Commonly used methods include the multi-factor superposition method (35), the Scenic Beauty Evaluation (SBE) method (36, 37), the Semantic Differential (SD) method (38), the Analytic Hierarchy Process (AHP), and the Fuzzy Comprehensive Evaluation (FCE) method, all of which are widely applied to construct index systems for spatial suitability assessment (39). This study focuses on evaluating the suitability of the Cangzhou section of the Grand Canal for outdoor exercise and fitness activities. Specifically, it assesses whether the selected sample spaces are appropriate for such activities and provides a comprehensive evaluation of their degree of suitability. By integrating theoretical analysis with field investigations, this study adopts the Analytic Hierarchy Process (AHP) and the multi-factor composite index method as its principal methodological approaches, taking into account the specific characteristics of the Cangzhou section of the Grand Canal.

## 3 Research framework and methodology

### 3.1 Research framework

**Figure 1:**
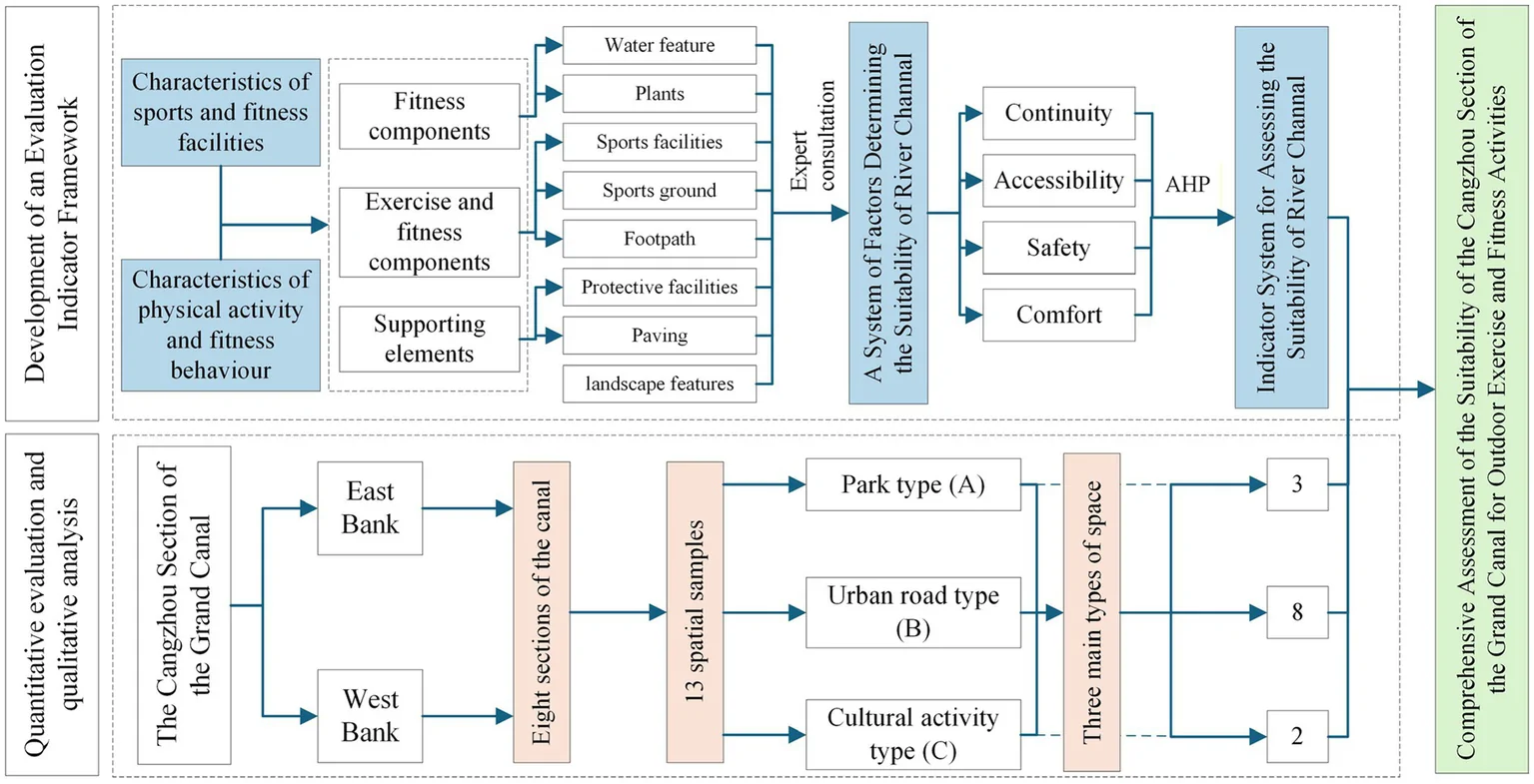
Research framework.

This study constructs a diagnostic framework for evaluating the suitability of urban canal spaces for physical exercise and identifying spatial differences among sample sites. The research process comprises four stages (Figure 1). First, the spatial requirements of physical exercise in blue-space and linear-corridor settings are identified through literature review and field investigation. Second, an evaluation framework is developed through expert consultation, with indicators organized under continuity, accessibility, safety, and comfort. Third, indicator weights are determined using AHP, while user questionnaires are used to assess the performance of the 13 sample sites. Finally, weighted suitability scores are compared across sites, banks, and spatial types to identify priority deficiencies and formulate differentiated planning implications.

### 3.2 Study area

**Figure 2:**
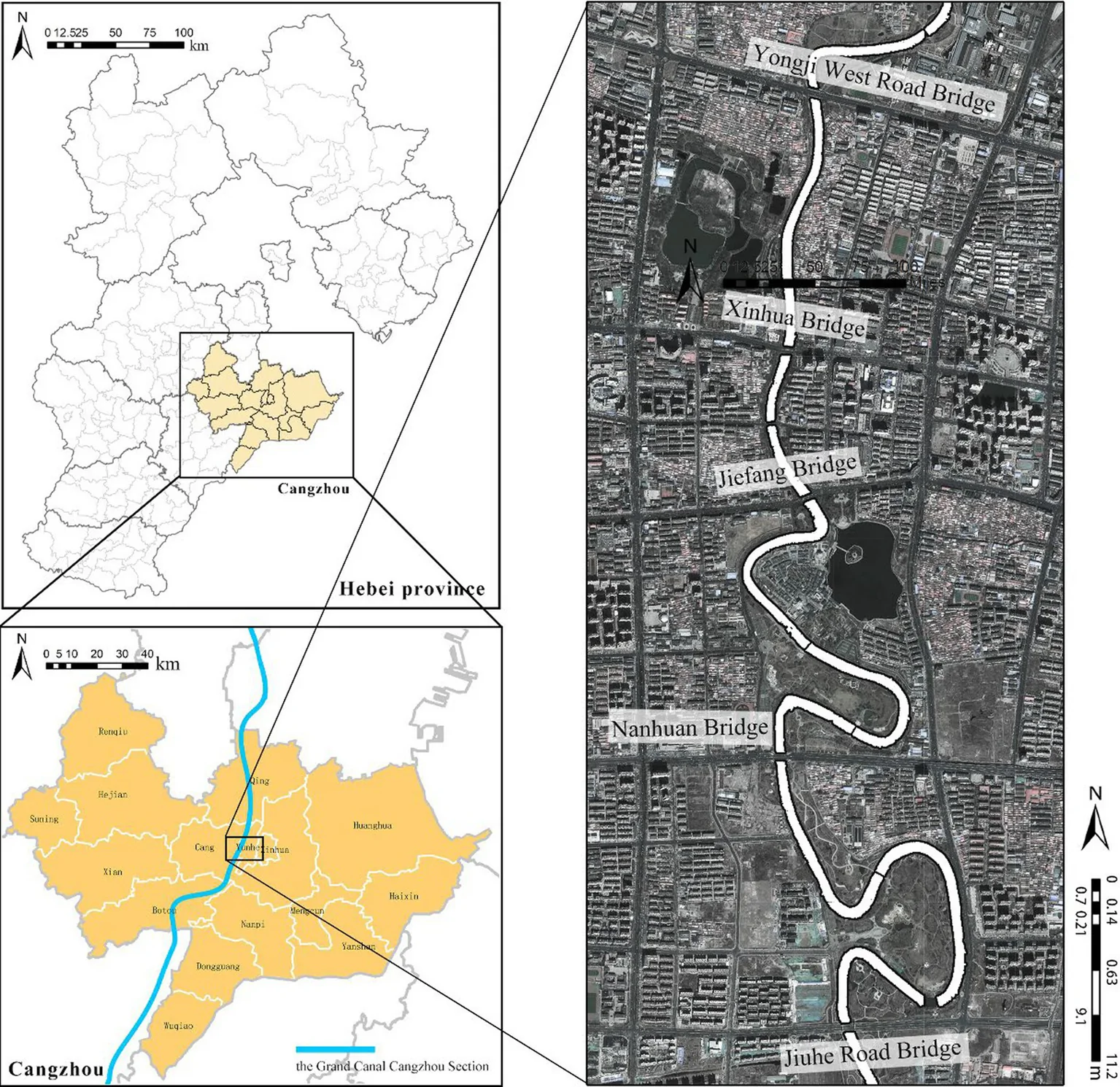
Study area.

The geographical Cangzhou section of the Grand Canal is approximately 215 km long, extending from Daxingzhuang Village in Wuqiao County to Li Youtun Village in Qing County and passing through Wuqiao County, Dongguang County, Nanpi County, Botou City, Cang County, the urban area of Cangzhou, and Qing County. This section accounts for approximately one-eighth of the Beijing–Hangzhou Grand Canal and serves as an important cultural, ecological, and recreational corridor in the region (Figure 2). The empirical investigation was restricted to a 24-km urban reach extending from Yongji Road in the north to Jiuhe Road in the south. The surveyed area included both banks and was divided into 13 sample sites according to bridge-defined segments, surrounding land-use characteristics, spatial interfaces, facility provision, and dominant public-space functions. The sites were classified into park-type, urban-road-type, and cultural-activity-type spaces (Tables 6, 7). The 13 sites were purposively selected to cover both canal banks, bridge-defined segments, and the three dominant spatial types identified within the urban reach. This maximum-variation sampling strategy was intended to capture spatial diversity within the surveyed reach rather than to provide statistical representation of the entire 215-km Cangzhou section.

**Table 6:** Classification of spatial types of river landscapes.

Reference	Type of space	Typical section		Definition
X	Park type	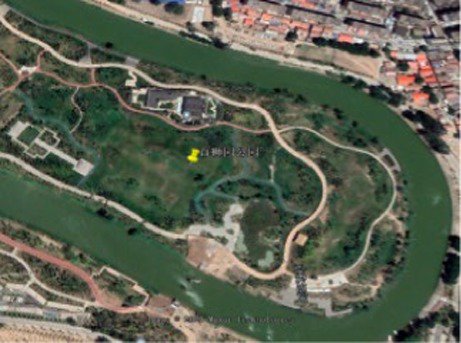	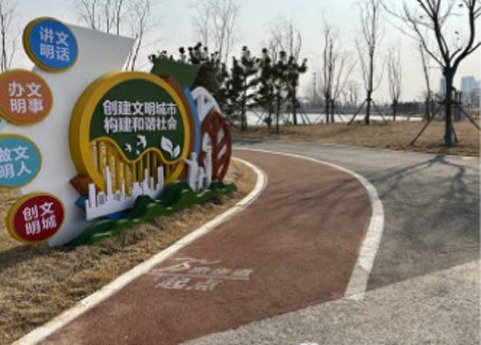	As part of the park’s green space network, it runs alongside the water’s edge.
Y	Urban road type	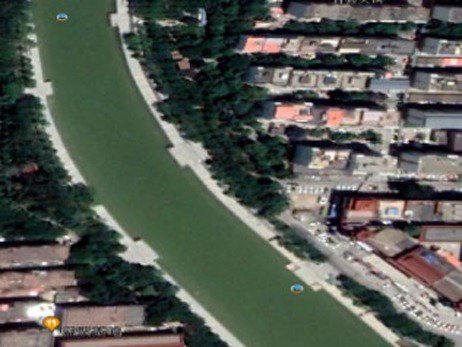	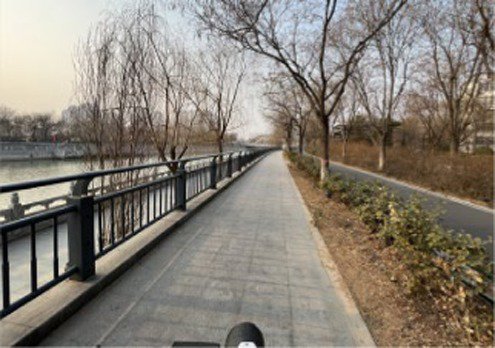	Separated from the urban road by a green belt, or running parallel to the pavement of the urban road.
Z	Cultural activities type	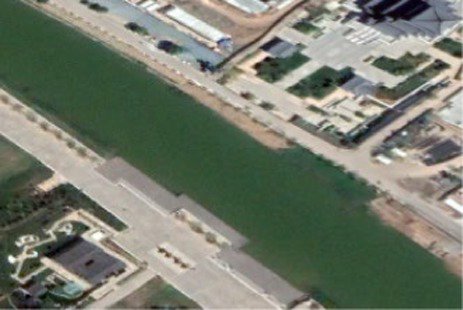	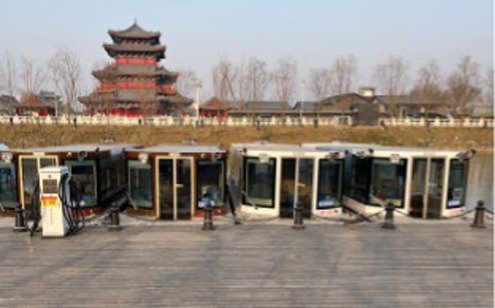	As part of the city’s key cultural spaces, it is situated on the waterfront.

**Table 7:** Sample spatial statistics.

Segmentation of river channel space		Reference	Spatial sample type
West Bank	Yongji West Road Bridge-Xinhua Bridge (1.38 km)	Y-1	Urban road type
	Xinhua Bridge-Rainbow Bridge-Jiefang Bridge (0.8 km)	Y-2	Urban road type
	Jiefang Bridge-Nanhuan Bridge (2.88 km)	Z-1	Cultural activities type
		Y-3	Urban road type
		X-1	Park type
	Nanhuan Bridge-Jiuhe Road Bridge (3.3 km)	Y-4	Urban road type
		X-2	Park type
East Bank	Yongji West Road Bridge-Xinhua Bridge (1.38 km)	Y-5	Urban road type
	Xinhua Bridge-Rainbow Bridge-Jiefang Bridge (0.95 km)	Y-6	Urban road type
	Jiefang Bridge-Nanhuan Bridge (2.88 km)	Z-2	Cultural activities type
		Y-7	Urban road type
	Nanhuan Bridge-Jiuhe Road Bridge (3.3 km)	X-3	Park type
		Y-8	Urban road type

### 3.3 Methodology

This study adopts AHP as the core methodological framework, complemented by field surveys, expert consultation, questionnaire surveys, and the multi-factor composite index method. The specific procedures are as follows:

Construction of the Hierarchical Model. The physical-exercise suitability of the river channel space in the Cangzhou section of the Grand Canal is defined as the objective layer (A). The evaluation criteria representing physical-exercise suitability constitute the criterion layer (B), while the specific influencing factors are assigned to the indicator layer (C).

Construction of the Judgment Matrix. To reduce subjectivity and arbitrariness in the evaluation process, the expert consensus method was employed. Experts were consulted through questionnaires, in which indicators and factors at different levels were compared pairwise to determine their relative importance. Based on these comparisons, judgment matrices were constructed. At the objective level, pairwise comparisons among elements within the comprehensive evaluation layer were conducted to generate the corresponding judgment matrix.

Determination of Factor Weights. The judgment matrices were solved using the eigenvalue method to derive the relative weights of elements under each criterion, referred to as single-level ranking. This process involves calculating the principal eigenvector of each judgment matrix and normalizing it to obtain the relative weights of factors at the same level with respect to the upper level.

Aggregation of Expert Judgments. The same panel of 30 experts participated in all three consultation rounds. The first two rounds focused on reviewing, screening, and refining the evaluation indicators, while the third round focused on pairwise comparisons of the retained criteria and indicators. The individual judgments were collated and revised through expert consensus, and a single group judgment matrix was established for each hierarchical level before weight derivation. The resulting group judgment matrices were entered into Yaahp, and the priority weights were calculated using the principal eigenvector method.

Consistency Testing. The consistency index (CI) and consistency ratio (CR) were calculated for the criterion-level and indicator-level judgment matrices. The CR values were 0.0923 for the criterion-layer matrix, 0.0000 for the continuity matrix, 0.0838 for the accessibility matrix, 0.0703 for the safety matrix, and 0.0553 for the comfort matrix. All CR values were below the threshold of 0.10, indicating that the judgment matrices satisfied the consistency requirements and were suitable for subsequent weight calculation. The consistency index and the largest eigenvalue were calculated using Equation 1 and 2, respectively.

Equation for calculating the consistency index:

$$\mathrm{CI}=\frac{\lambda_{\max}-n}{n-1}$$ (1)

Equation for calculating the largest eigenvalue $\lambda_{\max}$ :

$$\lambda_{\max}=\frac{1}{n}\sum_{i=1}^{n}\frac{{\left(\mathrm{AW}\right)}_{i}}{w_{i}}$$ (2)

The consistency ratio was calculated as CR = CI/RI, where RI is the random consistency index corresponding to the matrix order.

Hierarchical Calculation of Suitability Scores. A single calculation procedure was used throughout the study. For each site, the mean score of each questionnaire indicator was first calculated. Criterion-level scores were then obtained using the local AHP weights of the indicators within the corresponding criterion. Finally, each site-level composite score was calculated using the four criterion weights. This procedure is equivalent to multiplying the 18 indicator means by their global AHP weights and summing the products.

Step 1: Calculation of Site-Level Indicator Means. For site i and indicator j, the indicator mean was calculated from all valid questionnaires collected at that site using Equation 3:

$${\bar{X}}_{\mathrm{ij}}=(1/n_{i})\sum_{r}1^{\mathrm{ni}}X_{\mathrm{ijr}}$$ (3)

In this formula, $X_{\mathrm{ij}}$ denotes respondent *r*’s rating of indicator *j* at site *i*, nᵢ is the number of valid questionnaires at site *i*, and ${\bar{X}}_{\mathrm{ij}}$ is the site-level mean score of indicator *j*.

Steps 2 and 3: Calculation of Criterion and Composite Scores. The criterion score B_ᵢk_ was calculated as the weighted sum of the indicator means within criterion k, using local indicator weights w_j|k_. The site-level composite score Sᵢ was then calculated by weighting the four criterion scores using criterion weights W_k_, as shown in Equation 4:

$$B_{\mathrm{ik}}=\sum_{j}\in J_{k}w_{j|k}\;{\bar{x}}_{\mathrm{ij}};S_{i}=\sum_{k=1}W_{k}B_{\mathrm{ik}}$$ (4)

The site was treated as the primary spatial evaluation unit. Bank-level, spatial-type-level, and overall suitability scores were calculated as the arithmetic means of the composite scores of the sites included in each group. Criterion-level pooled means were reported descriptively and were not used to generate a separate overall score. All calculations used unrounded values; displayed values were rounded to three decimal places.

### 3.4 Questionnaire survey and evaluation procedures

Based on the evaluation index system for the suitability of river channel spaces for physical exercise, the questionnaire contained 18 items corresponding to indicators C1–C18 in Table 8. Each item was rated on a five-point Likert scale: 1 = very unsuitable, 2 = unsuitable, 3 = moderate, 4 = suitable, and 5 = very suitable. The on-site survey was conducted at the 13 sample sites from April to July 2025 during 09:00–12:00 and 14:00–18:00. Participants were recruited through on-site intercept sampling and consisted of exercisers already using the surveyed sites. Fifty questionnaires were distributed at each site, producing 650 questionnaires, of which 632 were valid (97.2%). Site-specific sample sizes are reported in Supplementary Table S1. AHP was subsequently used to determine indicator weights; the judgment matrices were analyzed in Yaahp, normalized, and subjected to consistency testing.

**Table 8:** Metric weight sorting.

Principles	Relative weighting	Indicator	Relative weighting	Absolute weighting	Rank
Continuity B1	0.0793	Continuity of water feature spaces C1	0.8333	0.0661	7
		Continuity of footpath spaces C2	0.1667	0.0132	14
Accessibility B2	0.2805	Accessibility of water feature spaces C3	0.3339	0.0937	5
		Accessibility of sports grounds C4	0.1848	0.0518	8
		Accessibility of sports facilities C5	0.0383	0.0107	16
		Accessibility of ancillary facilities C6	0.3621	0.1016	4
		Accessibility of footpath spaces C7	0.0809	0.0227	12
Safety B3	0.5609	Safety of water features C8	0.0582	0.0327	9
		Plant safety C9	0.3190	0.1789	1
		Footpath safety C10	0.1424	0.0799	6
		Sports facility safety C11	0.2031	0.1139	3
		Sports ground safety C12	0.2411	0.1353	2
		Safety of ancillary facilities C13	0.0361	0.0203	13
Comfort B4	0.0793	Comfort of water feature spaces C14	0.3060	0.0243	11
		Rationality of sports grounds C15	0.3893	0.0309	10
		Comfort of the footpath C16	0.1190	0.0094	17
		Diversity of sports facilities C17	0.1411	0.0112	15
		Richness of spatial interfaces C18	0.0447	0.0035	18

Questionnaire Reliability and Validity. The questionnaire demonstrated acceptable internal consistency, with an overall Cronbach’s alpha coefficient of 0.733. The Kaiser–Meyer–Olkin (KMO) measure was 0.625, and Bartlett’s test of sphericity was statistically significant (*p* = 0.01), indicating that the questionnaire data exhibited sufficient inter-item correlation and were suitable for subsequent analysis. Content validity was further supported by three rounds of consultation with the same multidisciplinary panel of 30 experts, during which the relevance, clarity, and applicability of the evaluation indicators were reviewed and refined.

## 4 Development of the evaluation system and evaluation results

### 4.1 Construction and validation of the evaluation framework

Based on the evaluation objectives, the assessment framework for the suitability of the Cangzhou section of the Grand Canal for physical exercise and fitness is structured into three interrelated levels. The first level is the objective level, which comprises the composite index used for evaluation. The second level is the criterion level, which includes the criteria and indicators selected to achieve the evaluation objectives. The third level is the indicator level, which specifies the factors determining each evaluation criterion.

**Figure 3:**
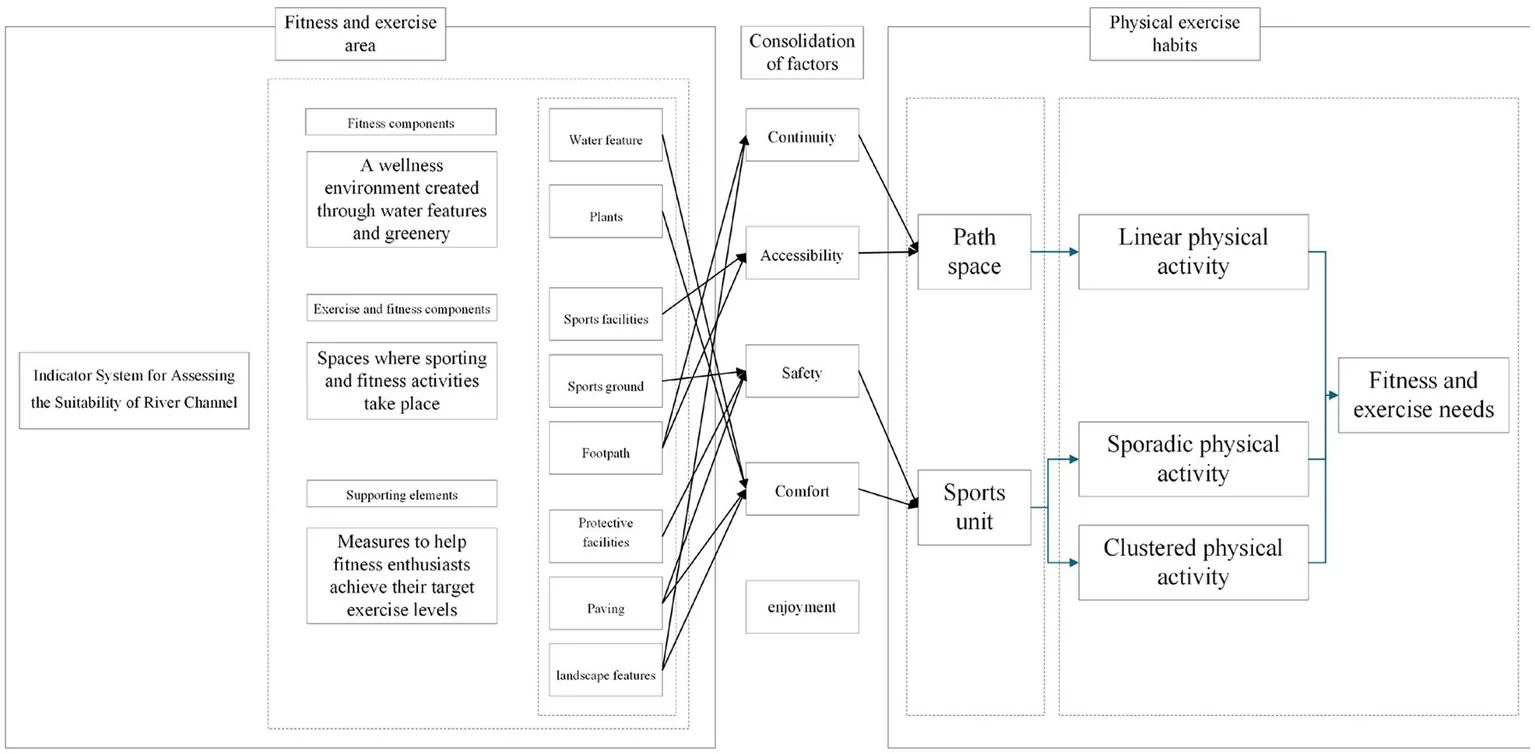
Logical analysis of exercise and fitness behavior and spatial action.

Based on the literature review and the definition of “suitability for exercise and fitness” proposed in this paper, the “types of exercise and fitness spaces” and the “characteristics of exercise and fitness behavior” in the Cangzhou section of the Grand Canal were integrated and synthesized to identify three key elements of exercise and fitness spaces: health and fitness elements, exercise and fitness elements, and auxiliary elements. Experts from multiple disciplines were invited to provide feedback on the evaluation indicators for exercise and physical-exercise suitability. After several rounds of consultation and refinement, “pleasure” was excluded, and “continuity,” “accessibility,” “safety,” and “comfort” were ultimately identified as the criterion-level factors (Figure 3).

#### 4.1.1 Selection of evaluation criteria

This study employed the expert consultation method to invite experts from multiple disciplines to contribute to the selection of indicators for evaluating the suitability of the Cangzhou section of the Grand Canal for physical exercise and fitness. After three rounds of evaluation, 20 relevant indicators were identified for assessing the suitability of the river channel spaces along this section for physical exercise and fitness. Accordingly, a preliminary evaluation framework consisting of an objective layer (A), a criterion layer (B), and an indicator layer (C) was established (Table 9).

**Table 9:** Evaluation index system of physical-exercise suitability of river landscape space.

Target (A)	Principles (B)	Indicator	Description of the metrics layer
Assessment of the Suitability of the Waterway Space along the Cangzhou Section of the Grand Canal for Physical Exercise	Continuity B1	Continuity of water feature spaces	The incorporation of water features in fitness spaces.
		Continuity of footpath spaces	Unimpeded access for people with mobility impairments.
	Accessibility B2	Accessibility of water feature spaces	The potential for water features in the space.
		Accessibility of sports grounds	The distinctiveness of the sports facilities and their appropriate integration with the road network.
		Accessibility of sports facilities	The layout of the sports and fitness facilities is well-designed and easy to navigate.
		Accessibility of ancillary facilities	The ancillary facilities are well-located and easy to spot.
		Accessibility of footpath spaces	The appropriate location of the footpath and its suitable connectivity.
	Safety B3	Safety of water features	The waterfront protection measures are well-designed, the revetment is fully functional, lifesaving equipment is in place, and warning signs are clearly visible.
		Plant safety	The venue is free of flying fluff, pollen and plants that are poisonous or have sharp, dangerous thorns, and the landscaping is designed to cater to the needs of those engaging in sports and fitness activities.
		Footpath safety	Installation of eco-friendly materials, signs of ageing, safety of the location.
		Sports facility safety	Is the installation safe? Are there any safety hazards? Is it in good condition and structurally sound? Are there any protective measures in place and are there instructions for use?
		Sports ground safety	Is the location safe? Is the construction in accordance with regulations? Is the maintenance carried out properly?
		Safety of ancillary facilities	Does the venue have rest areas, service facilities, lighting, emergency facilities and accessible facilities? Are the number and layout of signage appropriate, and are all facilities in good working order?
	Comfort B4	Comfort of water feature spaces	Does the site offer unobstructed views? Is there a wide variety of facilities for viewing, recreational activities and water-based experiences? Is the revetment design inviting? Does it allow for a variety of ways to engage with the water?
		Availability of sports facilities	Is there enough of it?
		Rationality of sports grounds	Is there a variety of spatial types? Are the individual units situated appropriately? Are the facilities comprehensive? Is the overall zoning plan well-structured?
		Comfort of the footpath	Are the pathways well-designed and unobstructed? Are their dimensions suitable for human use? Are they level?
		Diversity of sports facilities	Does the sports facility offer a wide variety of facilities to cater for all sports enthusiasts?
		Rhythm of the sports ground	Does the layout of the space and the coordination of details strike a balance between dynamic and static elements? Is the overall rhythm varied yet harmonious and cohesive?
		Display of cultural fabric	Structures integrated into the landscape, landscape features, or landscape elements that embody historical and cultural characteristics.

A total of 30 experts from the fields of landscape architecture, ecology, environmental psychology, and other related disciplines were invited to form an advisory panel, including 15 specialists in design, 3 in ecology, 2 in environmental psychology, 5 in sport science and 5 in human sciences. Thirty questionnaires were distributed and all were returned, yielding a response rate of 100%. The preliminary physical-exercise suitability evaluation indicators identified in the preceding section were then distributed to the experts to solicit their opinions and suggestions regarding the appropriateness of the selected indicators. The experts scored each indicator based on the characteristics of river channel space. Any indicator with an average score below 3.5 was removed. The selection frequency of each indicator was calculated according to the valid returned questionnaires (Table 10).

**Table 10:** Statistics of optimal results of evaluation indicators of exercise and physical-exercise suitability in river landscape space.

Evaluation criteria	Average score	Number of selections	Evaluation criteria	Average score	Number of selections
Continuity of water feature spaces C1	3.8	13	Footpath safety C10	4.8	27
Continuity of footpath spaces C2	4.5	24	Sports facility safety C11	5	30
Accessibility of water feature spaces C3	3.8	14	Sports ground safety C12	5	30
Accessibility of sports grounds C4	4.6	25	Safety of ancillary facilities C13	4.9	29
Accessibility of sports facilities C5	4.0	21	Comfort of water feature spaces C14	4.1	22
Accessibility of ancillary facilities C6	4.1	20	Rationality of sports grounds C15	4.3	21
Accessibility of footpath spaces C7	3.7	13	Comfort of footpaths C16	4.4	24
Safety of water features C8	4.2	22	Diversity of sports facilities C17	4.7	28
Plant safety C9	4.4	24	Availability of sports facilities	3.1	Eliminated
Richness of spatial interfaces C18 (Merged)	4.0	23	Rhythm of the sports ground C19 (merger)	3.6	12
			Display of cultural fabric C20 (merger)	3.5	11

After three rounds of expert consultation, 20 preliminary indicators were identified. “Availability of Sports Facilities” was excluded because its mean importance score was below the retention threshold. In addition, “Rhythm of Sports Ground” and “Display of Cultural Fabric” were merged into “Richness of Spatial Interfaces” because of conceptual overlap. The final framework therefore comprised 18 indicators, which were renumbered C1–C18 (Table 8).

#### 4.1.2 Establishment of an evaluation system

**Figure 4:**
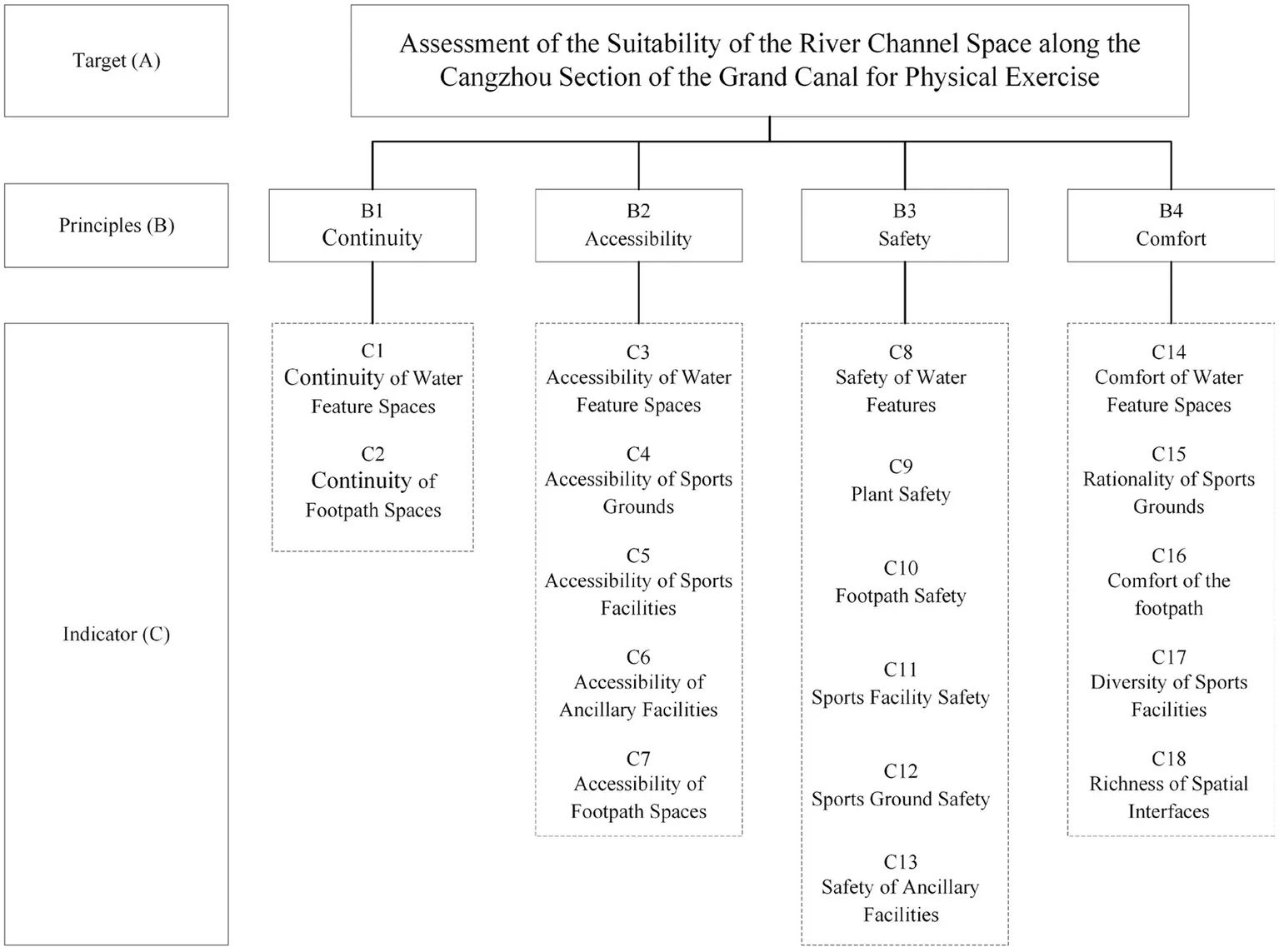
Spatial suitability assessment system of river channel spaces.

By applying scientific evaluation methods and principles, a river channel space suitability assessment framework has been established. The objective layer (A) comprises the assessment framework for the suitability of river channel spaces for physical exercise; the criteria layer (B1–B4) is divided into continuity, accessibility, safety and comfort; and the indicator layer (C1–C18) comprises a total of 18 assessment indicators (Figure 4). The linear character of the canal was primarily represented through indicators concerning the continuity of waterside spaces and footpaths, the accessibility of activity nodes along the corridor, and the safety and comfort of movement routes. These indicators were intended to evaluate whether individual spaces functioned as isolated recreational nodes or as components of a continuous exercise-supportive corridor.

### 4.2 Descriptive statistics of suitability scores

This study evaluated 13 sample areas. Based on the evaluation criteria established in the preceding section, 50 questionnaires were distributed in each section, for a total of 650 questionnaires. Of these, 632 were valid, yielding a valid response rate of 97.2%.

This paper divides the assessment of suitability for exercise and fitness into two components: a single-factor quality evaluation and an evaluation of the suitability of exercise and fitness in the river channel spaces of each segment and type. The single-factor quality evaluation is based on the average score of 18 factors, reflecting the specific elements that influence the suitability of the river channel spaces along the Cangzhou section of the Grand Canal for exercise and fitness. On this basis, the physical-exercise suitability of the river channel spaces along the Cangzhou section of the Grand Canal is further classified by segment and type, with suitability scores assigned to each segment and the river channel spaces within each segment ranked accordingly (Table 11).

**Table 11:** Comprehensive score value and grade comparison table.

Score	4.5≦Y≦5.0	4.0≦Y<4.5	3≦Y<4.0	2≦Y<3.0	1.0≦Y<2.0
Rating	I (Excellent)	II (Good)	III (Moderate)	IV (Poor)	V (Terrible)

The comprehensive scores obtained from the questionnaire survey of the sample spaces, as analyzed using the Analytic Hierarchy Process (AHP), indicate that notable score losses occurred across multiple single-factor quality assessments, with questionnaire scores falling below 3. Specifically, three items related to accessibility, three to safety, and one to comfort received relatively low scores, mainly concerning fitness and exercise elements. In contrast, nine single-factor quality indicators received relatively high feedback: four related to continuity, one to accessibility, and four to safety, primarily associated with the health and fitness elements within the space (Tables 12, 13).

**Table 12:** Questionnaire score statistics (West Bank).

Indicator	Y-1	Y-2	Z-1	Y-3	X-1	Y-4	X-2	Overall average
	Score	Score	Score	Score	Score	Score	Score	
Continuity of water feature spaces C1	4.7	4.2	4.2	4.3	4.8	4.5	4.8	4.500
Continuity of footpath spaces C2	4.4	4.8	4	3.6	4.8	3.2	4.8	4.229
Accessibility of water feature spaces C3	3.7	4.5	3.4	2.6	4.6	3.6	4.7	3.871
Accessibility of sports grounds C4	3	4.2	4.1	1	4.6	3.2	4.6	3.529
Accessibility of sports facilities C5	2.4	1	4.5	1	4.8	1	4.4	2.729
Accessibility of ancillary facilities C6	4.1	3.6	4	1	4.7	3	4.3	3.529
Accessibility of footpath spaces C7	4.5	4.7	4.5	1	4.4	2.3	4.4	3.686
Safety of water features C8	4.6	5	4.6	4.6	4.2	2.1	4.2	4.186
Plant safety C9	4.6	4.7	4	2.8	4.8	4.1	4.3	4.186
Footpath safety C10	3.8	3.6	4.5	2.2	4.4	3.2	4.6	3.757
Sports facility safety C11	1	2	4.6	1	4.1	1	4.3	2.571
Sports ground safety C12	1	1	4.5	2	4.2	2.2	4.1	2.714
Safety of ancillary facilities C13	3.8	3	4.4	1	4.1	2.1	4	3.200
Comfort of water feature spaces C14	3.2	2.6	2.2	2.6	4.3	3.3	4.3	3.214
Rationality of sports grounds C15	3.2	3	4.5	2.6	4.4	2.4	4.4	3.500
Comfort of the footpath C16	2.5	2.6	4.5	2	4.4	3.3	4.3	3.371
Diversity of sports facilities C17	2	2.2	4.6	1	4.3	2.6	4.6	3.043
Richness of spatial interfaces C18	3.4	3	4	2.2	4.6	2.4	4.4	3.429

**Table 13:** Questionnaire score statistics (East Bank).

Indicator	Y-5	Y-6	Z-2	Y-7	X-3	Y-8	Overall average
	Score	Score	Score	Score	Score	Score	
Continuity of water feature spaces C1	4.7	4.4	4.5	4.6	4.6	4.5	4.550
Continuity of footpath spaces C2	4.4	4.2	4	4	4.8	3.9	4.217
Accessibility of water feature spaces C3	3.7	4.6	4.3	3.2	4.4	4.2	4.067
Accessibility of sports grounds C4	3.6	2	2.2	2.1	4.5	4.2	3.100
Accessibility of sports facilities C5	1	1	1	1	4.4	3.4	1.967
Accessibility of ancillary facilities C6	3.6	2.2	2	2.2	4	3.2	2.867
Accessibility of footpath spaces C7	3.3	2.3	3.8	3.4	4.5	2.3	3.267
Safety of water features C8	4.4	4.6	5	4.4	4.2	4.2	4.467
Plant safety C9	3	4.6	4.8	4	4.1	4	4.083
Footpath safety C10	3.2	3.2	3	2.3	4.8	3.8	3.383
Sports facility safety C11	1	1	1	1	4.6	4.5	2.183
Sports ground safety C12	2.4	2.4	3	2.3	4.8	3.6	3.083
Safety of ancillary facilities C13	2.6	3.4	3.8	3.2	4.4	3.2	3.413
Comfort of water feature spaces C14	3	2.3	3.2	3.1	4.6	3.4	3.267
Rationality of sports grounds C15	2.8	3.2	4	3.2	4.5	3.6	3.550
Comfort of the footpath C16	2.8	2.6	3.2	3	4.4	4.1	3.350
Diversity of sports facilities C17	1	1	1	1	4.3	3.2	1.917
Richness of spatial interfaces C18	3.1	3	3.4	4.2	4.4	4.2	3.717

In terms of overall scores and ratings, the west bank achieved a mean score of 3.545, slightly higher than the east bank (3.422). Across the 13 surveyed sites, four sites were classified as Grade II, five as Grade III, and four as Grade IV. These values were calculated using the same hierarchical AHP procedure at every level of aggregation (Table 14).

**Table 14:** Comprehensive score for the sample space principles layer.

Bank	Principles	Weighting	Y-1	Y-2	Z-1	Y-3	X-1	Y-4	X-2
West Bank	Continuity B1	0.0793	0.369	0.341	0.330	0.332	0.381	0.340	0.381
	Accessibility B2	0.2805	1.046	1.122	1.088	0.430	1.298	0.871	1.263
	Safety B3	0.5609	1.603	1.716	2.447	1.232	2.466	1.512	2.399
	Comfort B4	0.0793	0.234	0.215	0.300	0.181	0.346	0.223	0.348
	Total	1	3.253	3.394	4.166	2.175	4.490	2.945	4.390
	Rating		III	III	II	IV	II	IV	II
	Overall rating	III (Moderate): 3.545							

Bank	Principles	Weighting	Y-5	Y-6	Z-2	Y-7	X-3	Y-8
East Bank	Continuity B1	0.0793	0.369	0.346	0.350	0.357	0.367	0.349
	Accessibility B2	0.2805	0.984	0.821	0.817	0.720	1.201	1.025
	Safety B3	0.5609	1.427	1.736	1.858	1.533	2.517	2.221
	Comfort B4	0.0793	0.208	0.201	0.255	0.228	0.356	0.283
	Total	1	2.988	3.104	3.280	2.838	4.441	3.878
	Rating		IV	III	III	IV	II	III
	Overall rating	III (Moderate): 3.422						

### 4.3 Differences across evaluation dimensions

The AHP-weighted continuity scores were generally high (Table 15). Six sites were classified as Grade I (46.2%) and seven as Grade II (53.8%); no sites were classified as Grades III–V. The mean continuity score across the 13 surveyed sites was 4.473, corresponding to Grade II (good). These results indicate that the canal and its embankment routes provide a relatively strong structural basis for continuous movement, although site-specific maintenance and route connections still require improvement.

**Table 15:** Evaluation results of the spatial continuity.

Segmentation of river channel space	Score	Grade
Yongji West Road Bridge–Xinhua Bridge Y-1	4.650	I
Jiefang Bridge–Nanhuan Bridge Z-1	4.167	II
Xinhua Bridge–Rainbow Bridge–Jiefang Bridge Y-2	4.300	II
Jiefang Bridge–Nanhuan Bridge Y-3	4.183	II
Jiefang Bridge–Nanhuan Bridge X-1	4.800	I
Nanhuan Bridge–Jiuhe Road Bridge Y-4	4.283	II
Nanhuan Bridge–Jiuhe Road Bridge X-2	4.800	I
Yongji West Road Bridge–Xinhua Bridge Y-5	4.650	I
Xinhua Bridge–Rainbow Bridge–Jiefang Bridge Y-6	4.367	II
Jiefang Bridge–Nanhuan Bridge Z-2	4.417	II
Jiefang Bridge–Nanhuan Bridge Y-7	4.500	I
Nanhuan Bridge–Jiuhe Road Bridge X-3	4.633	I
Nanhuan Bridge–Jiuhe Road Bridge Y-8	4.400	II

Accessibility showed greater variation across the surveyed sites (Table 16). Two sites were classified as Grade I (15.4%), two as Grade II (15.4%), five as Grade III (38.5%), three as Grade IV (23.1%), and one as Grade V (7.7%). The mean AHP-weighted accessibility score was 3.479, corresponding to Grade III (moderate). Field observations suggested that insufficient provision and uneven distribution of exercise grounds, facilities, ancillary services, and connected footpaths may have contributed to the lower scores at several sites.

**Table 16:** Evaluation results of the accessibility.

Segmentation of river channel space	Score	Grade
Yongji West Road Bridge–Xinhua Bridge Y-1	3.730	III
Jiefang Bridge–Nanhuan Bridge Z-1	3.878	III
Xinhua Bridge–Rainbow Bridge–Jiefang Bridge Y-2	4.001	II
Jiefang Bridge–Nanhuan Bridge Y-3	1.534	V
Jiefang Bridge–Nanhuan Bridge X-1	4.628	I
Nanhuan Bridge–Jiuhe Road Bridge Y-4	3.104	III
Nanhuan Bridge–Jiuhe Road Bridge X-2	4.501	I
Yongji West Road Bridge–Xinhua Bridge Y-5	3.510	III
Xinhua Bridge–Rainbow Bridge–Jiefang Bridge Y-6	2.927	IV
Jiefang Bridge–Nanhuan Bridge Z-2	2.912	IV
Jiefang Bridge–Nanhuan Bridge Y-7	2.567	IV
Nanhuan Bridge–Jiuhe Road Bridge X-3	4.282	II
Nanhuan Bridge–Jiuhe Road Bridge Y-8	3.654	III

Safety also varied across the surveyed sites (Table 17). Four sites were classified as Grade II (30.8%), four as Grade III (30.8%), and five as Grade IV (38.5%). No consistent descriptive pattern was observed across the three spatial types. The mean AHP-weighted safety score was 3.383, corresponding to Grade III (moderate). Lower scores were mainly associated with sports-facility safety, sports-ground safety, footpath conditions, and incomplete ancillary support.

**Table 17:** Evaluation results of the safety.

Segmentation of river channel space	Score	Grade
Yongji West Road Bridge–Xinhua Bridge Y-1	2.858	IV
Jiefang Bridge–Nanhuan Bridge Z-1	4.363	II
Xinhua Bridge–Rainbow Bridge–Jiefang Bridge Y-2	3.059	III
Jiefang Bridge–Nanhuan Bridge Y-3	2.196	IV
Jiefang Bridge–Nanhuan Bridge X-1	4.396	II
Nanhuan Bridge–Jiuhe Road Bridge Y-4	2.695	IV
Nanhuan Bridge–Jiuhe Road Bridge X-2	4.278	II
Yongji West Road Bridge–Xinhua Bridge Y-5	2.545	IV
Xinhua Bridge–Rainbow Bridge–Jiefang Bridge Y-6	3.096	III
Jiefang Bridge–Nanhuan Bridge Z-2	3.313	III
Jiefang Bridge–Nanhuan Bridge Y-7	2.733	IV
Nanhuan Bridge–Jiuhe Road Bridge X-3	4.487	II
Nanhuan Bridge–Jiuhe Road Bridge Y-8	3.959	III

Comfort was generally weaker than continuity and showed marked site-level variation (Table 18). Three sites were classified as Grade II (23.1%), three as Grade III (23.1%), and seven as Grade IV (53.8%); no sites were classified as Grades I or V. The mean AHP-weighted comfort score was 3.277, corresponding to Grade III (moderate). The principal deficiencies concerned the rationality and diversity of sports facilities, footpath comfort, and the quality of activity-supportive spatial interfaces.

**Table 18:** Evaluation results of the comfort.

Segmentation of river channel space	Score	Grade
Yongji West Road Bridge–Xinhua Bridge Y-1	2.956	IV
Jiefang Bridge–Nanhuan Bridge Z-1	3.788	III
Xinhua Bridge–Rainbow Bridge–Jiefang Bridge Y-2	2.717	IV
Jiefang Bridge–Nanhuan Bridge Y-3	2.285	IV
Jiefang Bridge–Nanhuan Bridge X-1	4.364	II
Nanhuan Bridge–Jiuhe Road Bridge Y-4	2.811	IV
Nanhuan Bridge–Jiuhe Road Bridge X-2	4.386	II
Yongji West Road Bridge–Xinhua Bridge Y-5	2.621	IV
Xinhua Bridge–Rainbow Bridge–Jiefang Bridge Y-6	2.534	IV
Jiefang Bridge–Nanhuan Bridge Z-2	3.210	III
Jiefang Bridge–Nanhuan Bridge Y-7	2.880	IV
Nanhuan Bridge–Jiuhe Road Bridge X-3	4.486	II
Nanhuan Bridge–Jiuhe Road Bridge Y-8	3.569	III

### 4.4 Type-based differences and overall suitability

#### 4.4.1 Differences among spatial types

**Figure 5:**
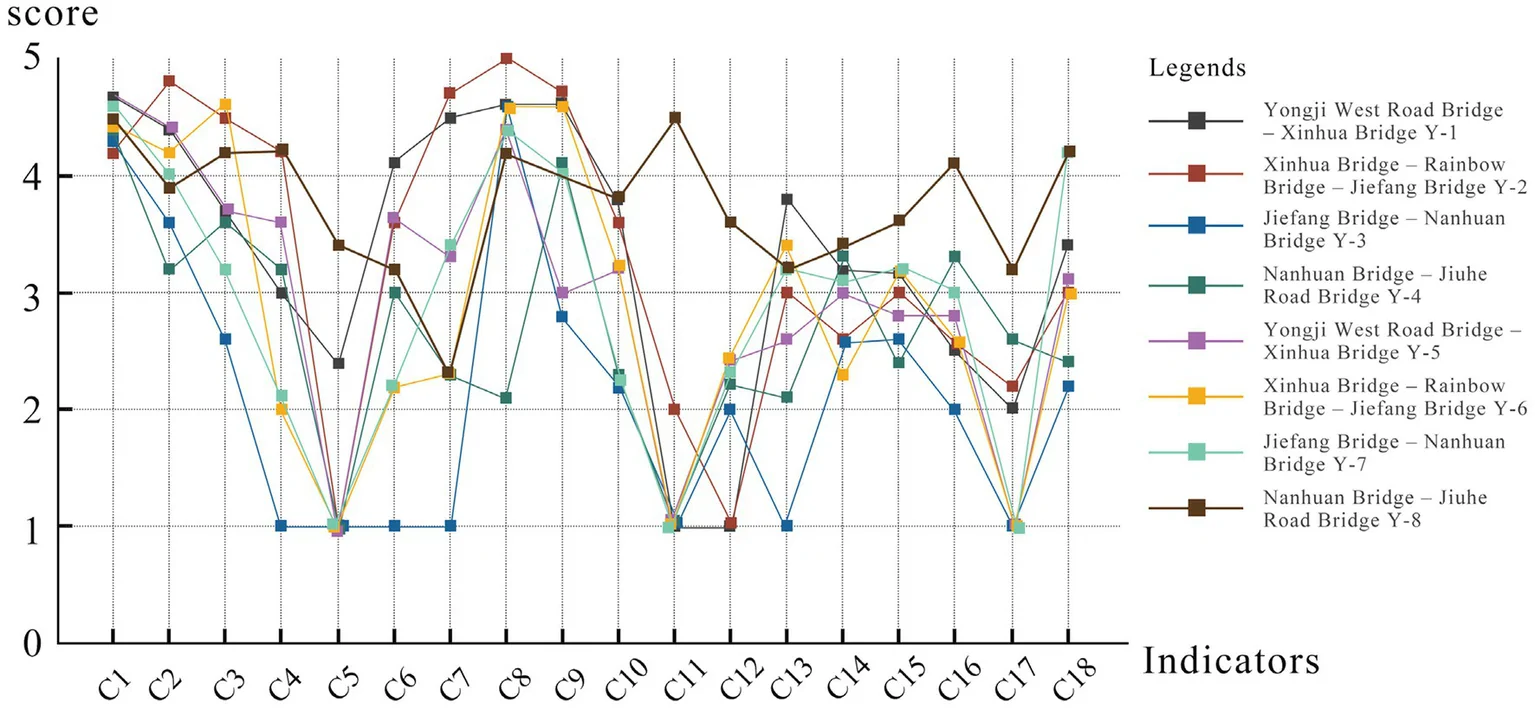
Indicator distribution map of physical-exercise suitability of urban road type river channel space.

Figure 5 provides a clear illustration of the distribution characteristics of single-factor quality in urban road-type river channel spaces, which can be summarized as follows: (1) The Y-1 section (Yongji West Road Bridge-Xinhua Bridge), Y-2 section (Xinhua Bridge-Jiefang Bridge), Y-6 section (Xinhua Bridge-Jiefang Bridge) and Y-8 section (Nanhuan Bridge-Jiuhé Road Bridge) exhibited marked variation in indicator scores in single-factor quality distribution, with overall suitability for exercise and fitness rated as moderate; (2) The Jiefang Bridge-Nanhuan Bridge Y-3 section, the Nanhuan Bridge–Jiuhe Road Bridge Y-4 section, the Yongji West Road Bridge-Xinhua Bridge Y-5 section, and the Jiefang Bridge-Nanhuan Bridge Y-7 section generally score low across all factors, resulting in a lower overall spatial quality; (3) The main issues with this type of river channel space center on the absence of elements conducive to exercise and fitness, as well as inadequate maintenance; in some cases, this is further compounded by insufficient accessibility of the footpaths, which hinders the development of walking and running activities. The overall suitability of urban road-type river channel spaces for physical exercise is classified as Grade III. A total of four sections are rated as Grade IV (poor), while the highest-scoring section, Nan’an Bridge–Jiuhe Road Bridge Y-8, reaches Grade III (moderate). Overall, the suitability of these spaces for physical exercise remains relatively low. These findings indicate an urgent need for future optimization and targeted renovation to improve their capacity to support exercise and fitness activities (Table 19).

**Table 19:** Comprehensive score of physical-exercise suitability of urban road type river channel space.

Principles	weighting	Y-1	Y-2	Y-3	Y-4	Y-5	Y-6	Y-7	Y-8
Continuity B1	0.0793	0.369	0.341	0.332	0.340	0.369	0.346	0.357	0.349
Accessibility B2	0.2805	1.046	1.122	0.430	0.871	0.984	0.821	0.720	1.025
Safety B3	0.5609	1.603	1.716	1.232	1.512	1.427	1.736	1.533	2.221
Comfort B4	0.0793	0.234	0.215	0.181	0.223	0.208	0.201	0.228	0.283
Total	1	3.253	3.394	2.175	2.945	2.988	3.104	2.838	3.878
Rating		III	III	IV	IV	IV	III	IV	III
Overall rating	III (Moderate): 3.072								

**Figure 6:**
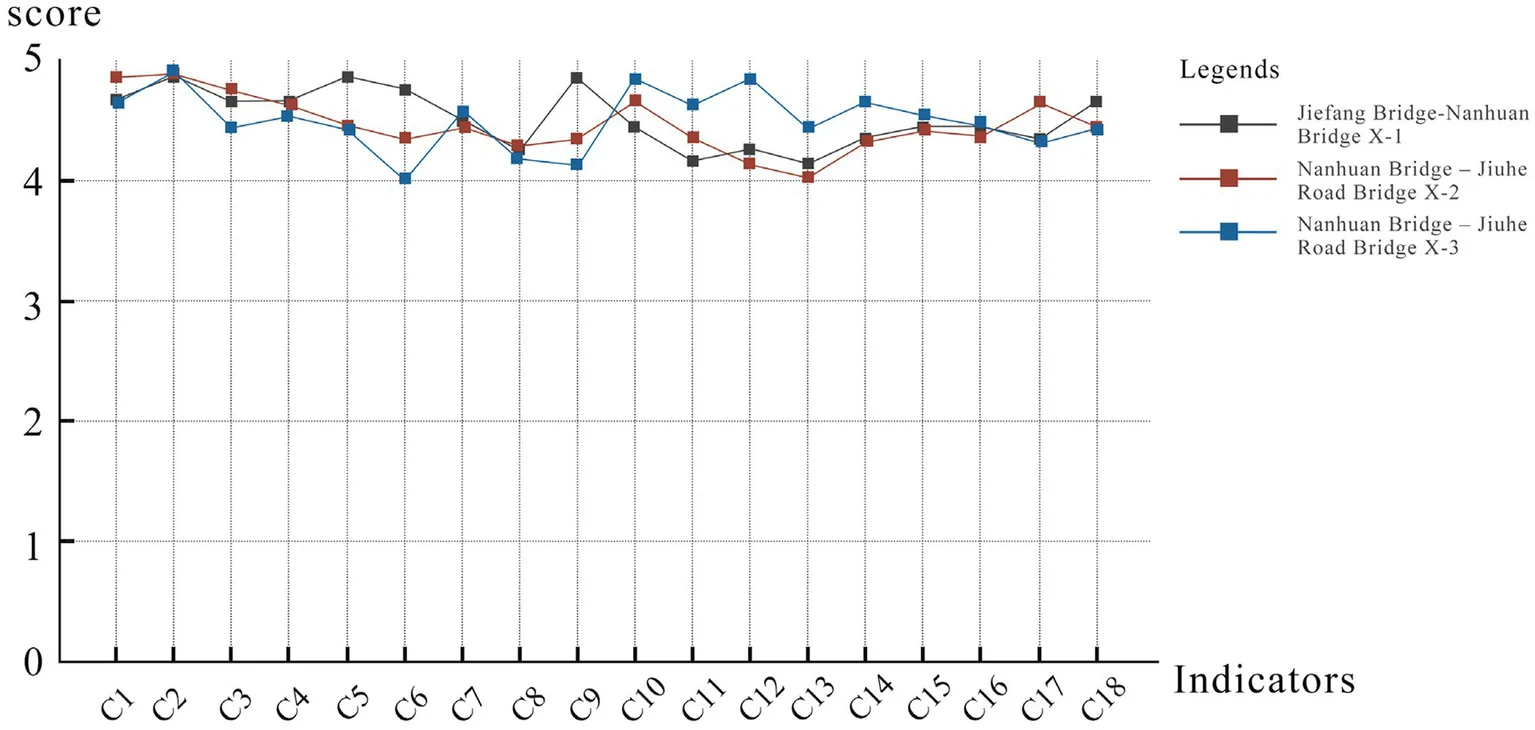
Indicator distribution map of physical-exercise suitability of park type river channel space.

Figure 6 illustrates the distribution characteristics of single-factor quality in park-style river channel spaces, which can be summarized as follows: (1) In the three sample sections-Section X-1, Section X-2, and Section X-3 (all extending from Nanhuan Bridge to Jiuhé Road Bridge)-the scores for all evaluated factors are generally high, indicating stable and overall good quality conditions. (2) Across the park-style river channel spaces, there is limited variation among sections, and the identified issues are largely consistent. The two most prominent problems are insufficient planning and maintenance of ancillary facilities, and inadequate safety conditions of sports and fitness areas and related equipment. Overall, the performance has reached Grade II standards; however, there remains room for further improvement (Table 20).

**Table 20:** Comprehensive score of physical-exercise suitability of park type river channel space.

Principles	Weighting	X-1	X-2	X-3
Continuity B1	0.0793	0.381	0.381	0.367
Accessibility B2	0.2805	1.298	1.263	1.201
Safety B3	0.5609	2.466	2.399	2.517
Comfort B4	0.0793	0.346	0.348	0.356
Total	1	4.490	4.390	4.441
Rating		II	II	II
Overall rating	II (Good): 4.441			

**Figure 7:**
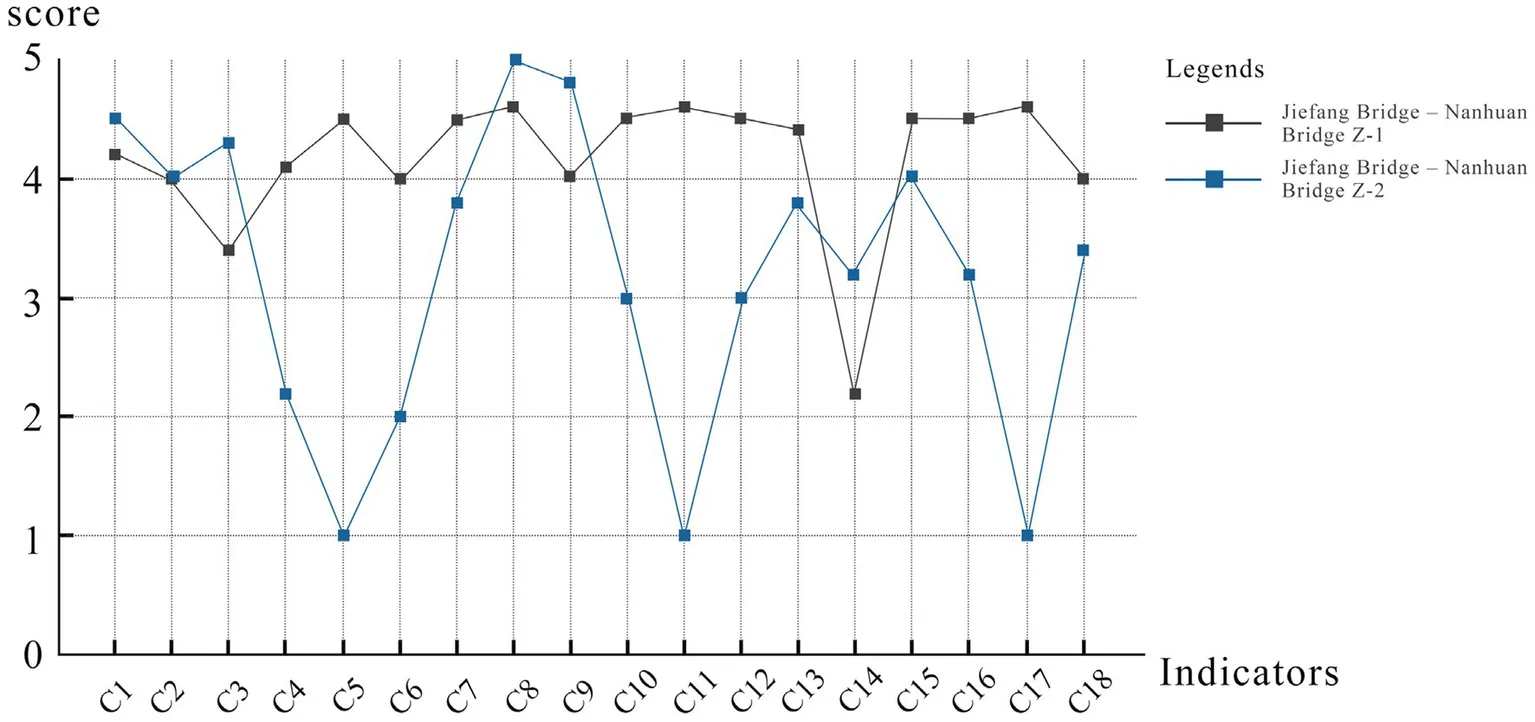
Indicator distribution map of physical-exercise suitability of cultural activity river channel space.

Figure 7 illustrates the distribution characteristics of single-factor quality in cultural activity-oriented river channel spaces. The main findings can be summarized as follows: (1) The Z-1 section between Jiefang Bridge and Nanhuan Bridge exhibits the most prominent overall performance in single-factor quality distribution. Except for a relatively low score in the safety of the C14 water feature space, the overall quality of this section is relatively high. (2) The single-factor quality distribution of the Z-2 section between Jiefang Bridge and Nanhuan Bridge shows considerable fluctuation. Influenced by indicators C5, C11, and C17, its overall performance is at an average grade. (3) The number of sample spaces for cultural activity-oriented river channel spaces is relatively limited, and deficiencies related to sports facilities represent the most prominent issue. Table 21 clearly shows that the overall comprehensive quality of river channel spaces designed for cultural activities meets Grade III standards.

**Table 21:** Comprehensive score of physical-exercise suitability of cultural activity type river channel space.

Principles	Weighting	Z-1	Z-2
Continuity B1	0.0793	0.330	0.350
Accessibility B2	0.2805	1.088	0.817
Safety B3	0.5609	2.447	1.858
Comfort B4	0.0793	0.300	0.255
Total	1	4.166	3.280
Rating		II	III
Overall rating	III (Moderate): 3.723		

#### 4.4.2 Overall assessment

As shown in Table 22, the site was used as the primary unit of aggregation. The arithmetic mean of the 13 hierarchically weighted site scores was 3.488, corresponding to Grade III (moderate). The surveyed sites displayed a clear imbalance among the four dimensions: continuity performed relatively well, whereas accessibility, safety, and comfort showed greater deficiencies. Park-type sites achieved the highest mean score (4.441), followed by cultural-activity-type sites (3.723) and urban-road-type sites (3.072).

**Table 22:** Cangzhou section canal river space sports physical-exercise suitability comprehensive score.

Target	Score	Grade	Principles	Principles		Weighting
				Average score	Grade	
Suitability for exercise and fitness	3.488	III (Moderate)	Continuity B1	4.473	II	0.0793
			Accessibility B2	3.479	III	0.2805
			Safety B3	3.383	III	0.5609
			Comfort B4	3.277	III	0.0793

## 5 Discussion

### 5.1 Key suitability patterns in the surveyed Urban Canal reach

Based on the evaluation of the 13 surveyed canal spaces, clear descriptive differences were observed among individual sites and spatial types. Indicators with scores of 4.0 or above, corresponding to Grades I and II, were regarded as relative strengths, whereas indicators scoring below 4.0 were identified as priorities for improvement. This classification was used to compare the spatial conditions supporting physical exercise across park-type, urban-road-type, and cultural-activity-type canal spaces.

One of the most important findings is the mismatch between the relatively high continuity score and the moderate overall exercise suitability of the surveyed canal spaces. The linear form of the canal and its embankment routes provides a basic physical structure for continuous movement. However, spatial continuity alone does not ensure that the corridor can effectively support regular physical exercise. Users also require convenient access points, safe and well-maintained paths, appropriate exercise grounds, supporting amenities, lighting, and comfortable environmental conditions. The results therefore suggest that the health-supportive value of a linear blue-space corridor depends on whether its structural continuity is translated into usable and activity-supportive continuity. This finding is consistent with previous studies showing that the presence or physical continuity of blue and green spaces does not automatically result in higher levels of physical activity (4). Actual use also depends on practical access, perceived safety, route quality, maintenance, and the availability of supporting facilities (40, 41). However, the present study extends these findings to a linear heritage canal context by showing that longitudinal continuity may coexist with substantial deficiencies in activity-supportive infrastructure (42).

The comparative assessment further showed that park-type canal spaces achieved the highest overall exercise-suitability scores among the three spatial types (Table 23). Their better performance was associated with relatively complete pedestrian networks, clearer functional organization, more concentrated exercise and recreational facilities, and stronger maintenance conditions. By contrast, urban-road-type and cultural-activity-type spaces were more frequently constrained by fragmented access, traffic interference, insufficient supporting facilities, and weak integration between activity routes and surrounding spatial resources. Park-type spaces may therefore provide a useful reference for the renewal of other canal-space types; however, their design features should be adapted rather than directly replicated, particularly in culturally sensitive heritage settings.

**Table 23:** Factor screening for different types of spaces.

Type of sample space	Key performance indicators	Potential indicators
Park type river channel space	C1, C2, C3, C4, C5, C6, C7, C8, C9, C10, C11, C12, C13, C14, C15, C16, C17, C18	None
Urban road type river channel space	C1, C2, C8, C9,	C3, C4, C5, C6, C7, C10, C11, C12, C13, C14, C15, C16, C17, C18
Cultural activity type river channel space	C1, C2, C7, C8, C9, C13, C15	C3, C4, C5, C6, C10, C11, C12, C14, C16, C17, C18

### 5.2 Priority–performance gaps in safety and accessibility

The evaluation revealed a mismatch between the importance of safety and accessibility and their actual performance. Safety received the highest expert-derived weight, but its score remained at a moderate grade. In canal-side spaces, safety involves not only protection from water-edge risks, but also pavement conditions, vegetation visibility, exercise equipment, lighting, and conflicts among pedestrians, bicycles, and motor vehicles. Field investigations showed that vehicle encroachment, insufficient lighting, and damaged surfaces were observed in sites with lower route-continuity and safety scores. Accessibility also remained moderate, indicating that the physical continuity of the canal did not necessarily translate into convenient use. Some sites contained continuous embankments but lacked legible entrances, unobstructed footpaths, and connections among exercise grounds, facilities, and surrounding urban spaces. The high priority assigned to safety and accessibility is consistent with previous assessments of parks and waterfront spaces, in which perceived risk, lighting, pavement quality, and access conditions were identified as major determinants of outdoor-space use (4, 43). Compared with bounded parks, however, canal corridors involve additional risks arising from water edges, extended routes, repeated road crossings, and conflicts among pedestrians, bicycles, and motor vehicles (44, 45).

Clear differences were also observed among the three spatial types. Park-type spaces achieved the highest suitability because they generally provided more complete pedestrian networks, clearer functional organization, better maintenance, and more concentrated recreational facilities. However, some park spaces remained weakly connected to surrounding streets and adjacent waterfront sections. Urban-road-type spaces showed the lowest suitability, mainly because of fragmented footpaths, traffic interference, insufficient facilities, and uneven maintenance. Cultural-activity-type spaces performed moderately. Although cultural landmarks enhanced spatial identity, they did not automatically improve exercise suitability because heritage resources, walking routes, and supporting facilities were not always effectively integrated.

These findings indicate that the exercise-supportive value of a linear blue-space corridor depends on whether structural continuity is converted into safe, accessible, and comfortable conditions for repeated use. Water presence, cultural resources, and continuous embankments provide spatial potential, but this potential can only be realized through appropriate route organization, risk control, facility provision, and maintenance.

### 5.3 Spatial-type differences and their underlying causes

For park-type spaces, optimization should prioritize connections between internal exercise routes and the surrounding urban environment. Hard boundaries such as roads and walls may be softened through vegetation and landscape interventions to create more continuous spatial interfaces and reduce psychological barriers to entry (46, 47). Materials, colors, and boundary treatments should also be coordinated with the canal landscape to strengthen visual continuity (48). At the same time, pedestrian–vehicle separation and parking management should be improved to prevent vehicles from occupying access paths (49, 50).

For urban-road-type spaces, the primary task is to establish continuous and safe pedestrian routes. Narrow or disconnected paths should be integrated and widened where possible, while obstacles and inappropriate occupation of walking areas should be removed. Lighting should be continuous and sufficient to support evening exercise (51), and damaged or slippery surfaces should be replaced with level and slip-resistant materials, particularly in areas frequently used by older adults and children (52). Comfort-oriented improvements, including vegetation, resting points, water views, and small-scale landscape elements, should be introduced only after basic accessibility and safety deficiencies have been addressed.

For cultural-activity-type spaces, exercise routes should be better connected with cultural landmarks and surrounding scenic areas. In the Z-2 section, for example, weak pedestrian connections and inadequate wayfinding reduced the integration between the canal-side trail and the Langyin Tower scenic area. Route organization, signage, materials, and colors should therefore respond to the historic context and incorporate cultural resources into continuous walking sequences. Where navigation, flood-control, and heritage-conservation requirements permit, low-impact waterside facilities such as viewing platforms, boardwalks, and ecological embankments may be introduced to strengthen interaction with the water and improve spatial vitality (53–55).

Overall, planning interventions should follow a clear priority sequence: first addressing safety and accessibility, then supplementing exercise and ancillary facilities, and finally improving environmental comfort and cultural experience. Differentiated interventions are preferable to applying a uniform park-style model to all canal spaces, particularly within heritage-sensitive environments.

### 5.4 Theoretical and planning implications

The findings suggest that physical-exercise suitability in linear heritage blue spaces depends not only on water presence or corridor continuity, but also on whether these spatial resources are translated into safe, accessible, and comfortable opportunities for repeated use. This extends conventional blue-space assessment by emphasizing practical usability and the integration of heritage resources with everyday physical activity.

From a planning perspective, interventions should follow a clear sequence: first improve safety and accessibility, then supplement exercise and ancillary facilities, and finally enhance environmental comfort and cultural experience. Park-type spaces should strengthen external connections, urban-road-type spaces should prioritize continuous and safe footpaths, and cultural-activity-type spaces should better integrate exercise routes with heritage landmarks and waterside experiences. This differentiated approach avoids applying a uniform park-style model to all canal spaces.

### 5.5 Limitations

This study has yielded several findings regarding the development of an evaluation system for assessing the suitability of the Cangzhou section of the Grand Canal for physical exercise and fitness. However, several limitations remain that should be addressed in future research.

(1) The construction of the evaluation system is still at an early stage. Limitations exist in the refinement of indicators, and evaluations based on user groups inevitably involve a degree of subjectivity. In addition, both research on river channel space suitability for exercise and fitness and the river channel spaces themselves are dynamic and evolving processes, requiring further interdisciplinary approaches to enrich and refine the evaluation content. (2) The investigation of river channel spaces has primarily focused on their physical spatial characteristics. Some of the surveyed sections were undergoing renovation during the study period, which had a limited impact on the evaluation results and introduced certain constraints on the robustness of the conclusions. Future studies should conduct more continuous and in-depth investigations of such sections to improve result reliability. (3) The 13 sites were selected to cover the principal spatial types within the surveyed 24-km urban reach; however, they cannot represent the full spatial diversity of the approximately 215-km Cangzhou section. The numerical results should therefore be interpreted as evidence from the surveyed urban reach rather than as an assessment of the entire Cangzhou section. Future studies should expand the sample to suburban and rural canal reaches and examine whether the framework performs consistently across different settlement and landscape contexts. (4) The proposed optimization strategies remain at a conceptual and directional grade and have not yet been translated into detailed planning and design guidelines. Future research should build on this foundation by further refining the evaluation framework and incorporating additional multidimensional indicators that integrate both subjective and objective dimensions, thereby improving and strengthening the suitability evaluation system for the Cangzhou section of the Grand Canal. (5) Respondents were recruited exclusively from exercisers who were already using the surveyed canal spaces during the survey periods. Residents who avoided or rarely used these spaces because of poor accessibility, safety concerns, inadequate facilities, or other unfavorable conditions were therefore not represented. This user-selection bias may have led to an overestimation of perceived suitability. Future studies should combine on-site user surveys with community- or household-based sampling of both users and non-users.

## 6 Conclusion

This study examined 13 sample sites within a 24-km urban reach of the Grand Canal in Cangzhou. Field surveys, user questionnaires, expert consultation, and AHP were used to construct and apply a context-adapted physical-exercise suitability framework. The main findings are as follows: (1) the study area was divided into 13 sample sites and classified into urban-road-type, park-type, and cultural-activity-type spaces; (2) four sites were rated as Grade II, five as Grade III, and four as Grade IV. The west bank achieved a mean score of 3.545, slightly higher than the east bank (3.422); (3) the mean criterion scores were 4.473 for continuity (Grade II), 3.479 for accessibility (Grade III), 3.383 for safety (Grade III), and 3.277 for comfort (Grade III); (4) park-type spaces achieved the highest mean suitability (4.441, Grade II), followed by cultural-activity-type spaces (3.723, Grade III) and urban-road-type spaces (3.072, Grade III); and (5) the mean composite suitability score of the 13 surveyed sites was 3.488, corresponding to Grade III. This result characterizes the surveyed urban reach and should not be generalized to the entire Cangzhou section.

## Data Availability

The datasets presented in this study can be found in online repositories. The names of the repository/repositories and accession number(s) can be found in the article/supplementary material.
